# A resected case of symptomatic acinar cell cystadenoma of the pancreas displacing the main pancreatic duct

**DOI:** 10.1186/s40792-016-0166-1

**Published:** 2016-04-23

**Authors:** Haruyoshi Tanaka, Tsuyoshi Hatsuno, Mitsuru Kinoshita, Kazuya Hasegawa, Hiromasa Ishihara, Nao Takano, Satofumi Shimoyama, Hiroshi Nakayama, Masato Kataoka, Shu Ichihara, Mitsuro Kanda, Yasuhiro Kodera, Ken Kondo

**Affiliations:** Department of Gastroenterological Surgery (Surgery II), Nagoya University, Tsurumai-cho 65, Syowa-ku, Nagoya, 466-8550 Japan; Department of Surgery, Nagoya Medical Center, National Hospital Organization, sannomaru 4-1-1, naka-ku, Nagoya, 460-0001 Japan; Department of Pathology, Nagoya Medical Center, National Hospital Organization, sannomaru 4-1-1, naka-ku, Nagoya, 460-0001 Japan

**Keywords:** Acinar cell cystadenoma, Pancreas, Cystic transformation, Symptomatic

## Abstract

Acinar cell cystadenoma (ACA) of the pancreas has been newly recognized as an entity by the World Health Organization (WHO) definition (2010), and its pathogenesis has not been known adequately because of the rarity. Here, we report a case of a 22-year-old female who had been followed up for a cystic lesion at the tail of the pancreas pointed out by a screening computed tomography (CT) scan 7 years ago. The tumor grew in size from 3.3 to 5.1 cm in diameter for 6 years (0.3 cm per year). Particularly, it rapidly grew up to 6.3 cm in the latest 3 months in concurrence with the emergence of epigastralgia. A contrasted CT scan revealed the irregularly formed, multilocular cystic tumor having thin septum and calcification. The intratumoral magnetic resonance imaging intensity in the T1 and T2 weighted images were low and high, respectively. No communications between the tumor and the main pancreatic duct (MPD) were found, but the tumor displaced the MPD. She underwent surgical resection because the tumor was growing, turned symptomatic, and it seemed difficult to be diagnosed correctly until totally biopsied. Spleen-preserved distal pancreatectomy was performed. It was pathologically diagnosed as ACA; the cyst was lined by cells with normal acinar differentiation; cuboidal cells with round, basally oriented nuclei and eosinophilic granules in its apical cytoplasm. The abdominal pain has disappeared, and no recurrences have been found during a 5-year follow-up. Clinicians are recommended to consider an ACA as one of differential diagnoses of cystic tumors of the pancreas to provide appropriate diagnostics and therapeutics.

## Background

Acinar cell neoplasms of the pancreas is a rare disease representing only 2 % of pancreatic neoplasms [1]. Acinar cystic transformation as a benign lesion, called an acinar cell cystadenoma (ACA), is particularly rare to be diagnosed. ACAs are benign cystic lesions lined by cells with acinar differentiation without atypical changes [2]. ACA was first reported as an autopsied case in April 2002 by Albores-Saavedra [3]. In the literature, 47 patients of ACAs of pancreas were reported so far [1, 3–13]. To date, the pathogenesis of ACAs remains unclear although it is assumed that it could be mainly acinar metaplasia [2]. The epidemiology and natural history of ACAs also remain unclear because of the insufficient number of reported cases. Additionally, ACAs confers difficulty in imaging diagnosis due to lack of distinctive features. Previous studies indicated that ACAs may harbor some genetic alterations. We herein reported a surgical case of ACA with bibliographic considerations.

## Case presentation

**Fig. 1:**
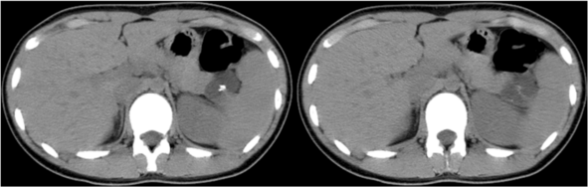
CT scan detected a low-dense and multilocular cystic tumor having thin septum and calcification. The tumor size is 3.3 cm in diameter

**Fig. 2:**
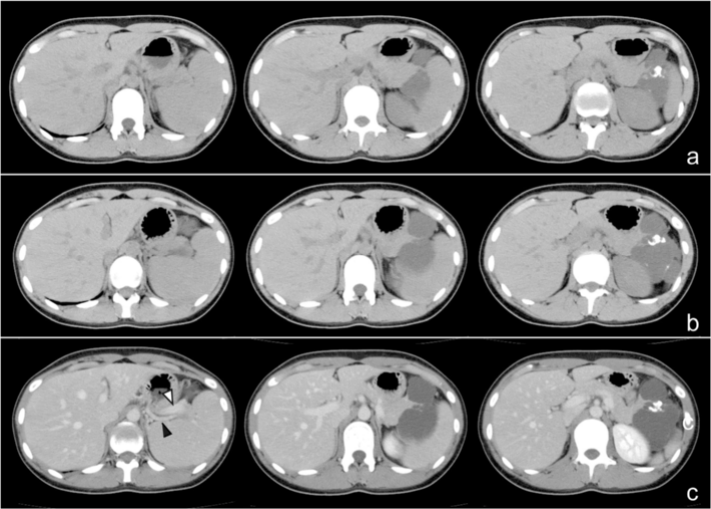
Enhanced CT scan showed that the tumor size increased to 5.1 cm in diameter for 6 years (**a**) and in the subsequent 3 months increased to 6.3 cm eventually (**b**). Its septum and capsule are not enhanced, and both splenic artery (*black arrow head*) and splenic vein (*white arrow head*) are not invaded by the tumor (**c**)

**Fig. 3:**
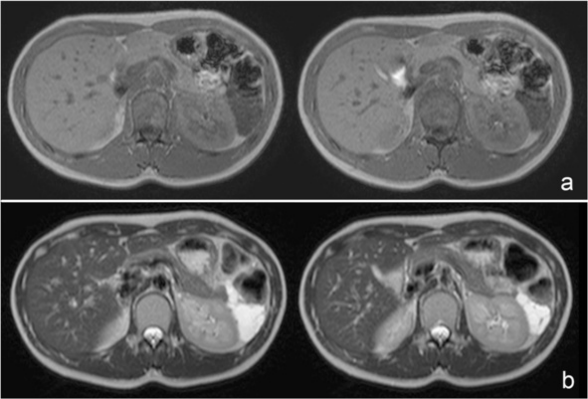
Magnetic resonance imaging revealed the mass low intensity in the T1-weighted image (**a**) and high intensity in the T2-weighted image (**b**)

**Fig. 4:**
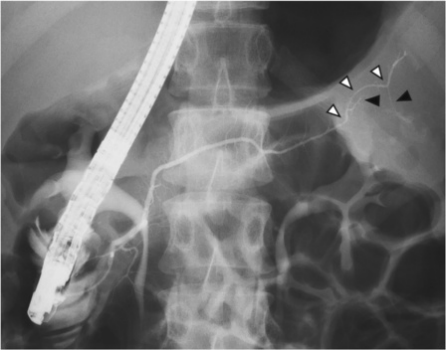
Endoscopic retrograde pancreatography showed that the distal portion of the main pancreatic duct is not stenosed but displaced by the tumor (*white arrow head*), and its branches are also clearly described (*black arrow head*)

A 22-year-old female had been followed up annually at a former hospital for a cystic mass at the tail of the pancreas, which had been pointed out by a screening computed tomography (CT) scan to diagnose an acute abdomen, such as peritonitis and ovarian torsion, when she was 15 years old (Fig. 1). After she has transferred to our hospital, a routine follow-up has been provided to put the lesion under surveillance. Six years after the first diagnosis, she started to suffer from intermittent epigastralgia and back pain usually at night, lasting for 2 to 3 h. The tumor grew in size from 3.3 to 5.1 cm in diameter for 6 years (0.3 cm per year). Particularly, it rapidly grew up to 6.3 cm in the last 3 months in concurrence with the emergence of epigastralgia (Fig. 2). A CT scan detected a solitary, irregularly formed, multilocular cystic tumor with thin septum and calcification. Its septum and capsule are not enhanced, and both splenic artery and vein are not invaded by the tumor (Fig. 2). In findings of magnetic resonance imaging (MRI), the tumor showed low intensity in T1 and high intensity in a T2 weighted image (Fig. 3). Endoscopic ultrasound sonography (EUS) identified a multilocular cystic lesion with calcification, but no nodules on the septal walls were detected. There were no communications between the cystic tumor and the main pancreatic duct (MPD) or any mucinous discharge from the papilla during endoscopic retrograde pancreatography (ERP). The distal portion of the MPD was clearly displaced by the tumor. Furthermore, small branches of the pancreatic duct around the tumor were detected (Fig. 4). Neither pancreatic juice cytology nor EUS-guided fine-needle aspiration were performed. She had no familial history, past medical history, or allergic history. Physical examination did not reveal any tenderness or palpable mass at the abdomen. The laboratory data before treatment was almost normal including tumor markers for pancreatic tumors.

Mucinous cystic neoplasm (MCN), serous cystic neoplasm (SCN), and branch duct intraductal papillary mucinous neoplasms (BD-IPMN) were considered as differential diagnoses. According to IPMN/MCN guidelines [14], surgical resection might be recommended because of her age and size of the tumor, being symptomatic and getting larger than before. Accordingly, we decided to propose surgical resection, and written informed consent was obtained.

**Fig. 5:**
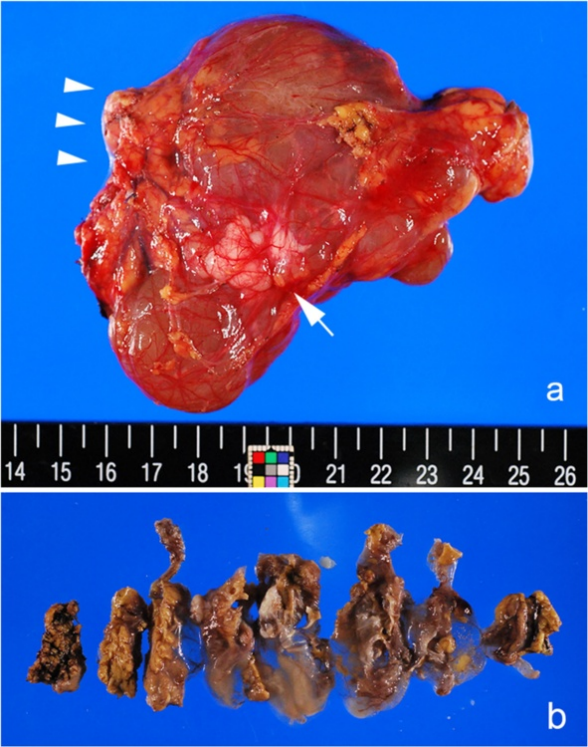
Macroscopic image of raw specimen (**a**) and fixated and cut specimen (**b**). The lesion is comprised of very soft and multilocular cysts with focal calcification (*arrow*). The cut margin is indicated by an *arrow head*

Intraoperatively, we found a very soft and multilocular cystic tumor having a thin capsule without adhesion or invasion to the adjacent tissues. Because the tumor was unlikely to be malignant, we performed a spleen-preserved distal pancreatectomy without a systemic lymph node dissection. Splenic artery and vein are also preserved. The cut line was easily determined because the tumor was well-demarcated macroscopically. Intraoperative ultrasonography also showed no evidence of mural nodules. We also ensured that the resection margins were cancer-free by examining intraoperative frozen sections. The lesion is comprised of very soft and multilocular cysts containing serous fluid and focal calcification (Fig. 5). The postoperative course was uneventful and she discharged on the postoperative day nine. After surgery, the abdominal pain has disappeared.

**Fig. 6:**
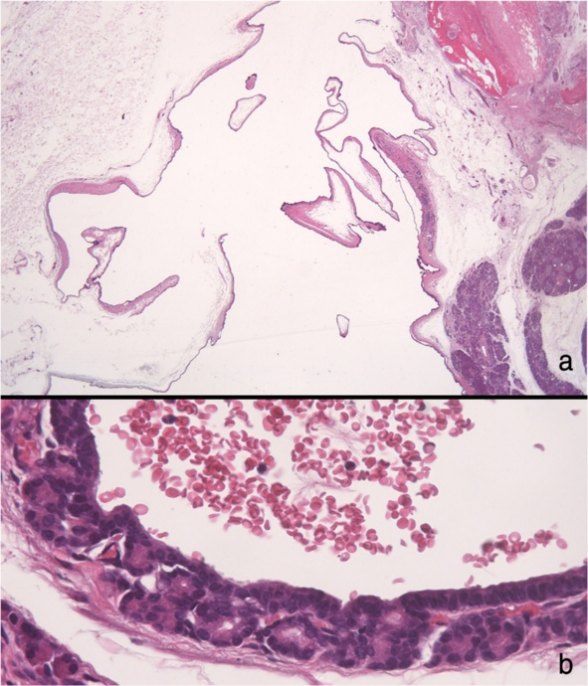
Image of microscopic specimen. Low magnified findings (**a**); the lesion is comprised of large and small cysts. High magnified findings (**b**); cystic lesion is lined by cells with acinar differentiation which have eosinophilic granules in their apical cytoplasms. The nuclei are uniform, lack atypia, and basally oriented

**Fig. 7:**
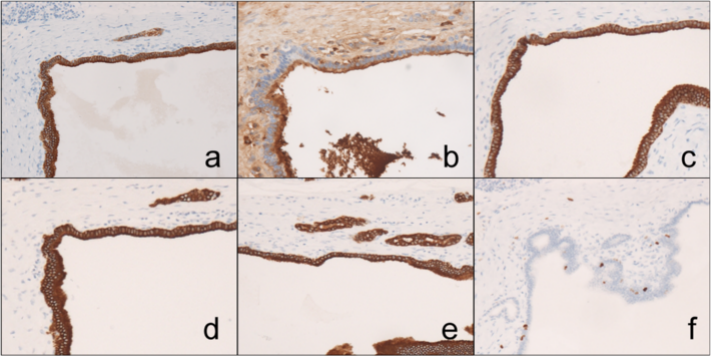
Immunohistochemical staining. Positive staining of α1-antichymotrypsin (**a**), α1-antitrypsin (**b**), AE1/AE3 (**c**), CAM-5.2 (**d**), and CK7 (**e**) were seen. The MIB-1 index is <1 % (**f**)

In pathological examinations, a cystic lesion lined by cells with acinar differentiation was pointed out. The nuclei are uniform and basally oriented without atypical changes (Fig. 6). In immunohistochemical staining, positive staining of α1-antichymotrypsin, α1-antitrypsin (pancreatic enzyme marker), AE1/AE3, CAM-5.2 and CK7 (epithelial marker), and MIB-1 index was <1 %. Based on these pathological findings, it was finally diagnosed as an ACA (Fig. 7). Five years have passed after resection, and no recurrences of the tumor and symptoms were seen.

### Discussion

Here, we reported a surgical case of an ACA of the pancreas, which showed interesting radiologic features and clinical courses. We have clarified the description of the MPD image, based on the results of ERP.

Acinar cell neoplasms are recognized as a rare entity. Particularly, ACAs, lined by cells with acinar differentiation without atypical changes, are reported to be extremely rare [2]. The acinar cells play a role in pancreatic exocrine. Although the acinar cells constitute a large portion of the pancreatic parenchyma, most malignant cells were derived from other components, such as ductal adenocarcinomas from the ductal cells and neuroendocrine tumors from the beta cells [2].

ACA incidentally detected during autopsy was first reported in April 2002 [3]. After that, several case reports have been reported, and eventually, the definition of ACA was established by the WHO classification of tumors of the digestive system in 2010 [15]. As of September 2015, 12 papers including 48 patients were identified in the literature search by the PubMed database (http://www.ncbi.nlm.nih.gov/pubmed) [1, 3–13]. All of them were diagnosed by the pathological findings of surgical or autopsied specimens.

In the literature review, females are predominant (30 of 48 cases, 64 %). Approximately two thirds of the patients had complains of an abdominal pain or discomfort. Postoperative follow-up period was widely ranged from 5 months to 11 years. There has not been any case reported on recurrence or evident malignant transformation so far. Whether ACAs is a congenital disease is dismissive so far because the age of the patients widely ranged 9–71 years old (mean 42.9 years). The most typical radiological appearances of ACAs are reported to be homogeneous hypovascular cystic tumors with thin septum. They show hypodensity in CT images, low intensity on the T1-weighted MRI, and high intensity on the T2-weighted MRI [13]. Usually, there are no communications between the cysts and the MPD.

However, it is hard to distinguish from other cystic tumors of the pancreas exclusively by imaging only because ACAs have less definitive morphologic features. The size of ACAs was reported to have a wide range from small incidentaloma to large tumor-suspected malignancy. Moreover, some ACAs diffusely involved entire pancreatic grand or increased in diameter during long-term follow-up. The most frequent site of ACAs was reported to be the head of the pancreas (around half of cases). Most of ACAs were solitary but some (approximately 10 %) of them have diffuse disease. The prevalence of unilocular or multilocular forms was equivalent. Less than 10 % of ACAs showed a communication to the MPD like BD-IPMNs. Eight cases of them are reported to have calcifications inside the tumor, and it is known to be a similar finding as SCNs. Some of them have thick capsules, which is frequently seen in MCNs. In the literature, pre-operative diagnoses are IPMN (eight cases), MCN (three cases), SCN (one case), and neuroendocrine tumor (one case), etc.

Although even EUS-guided fine-needle aspiration was performed in 21 cases, none of them were diagnosed as ACAs because of its hypocellularity [7]. Accordingly, it would be challenging to distinguish ACAs from other pancreatic cystic tumors by a pre-operative imaging. A review of the literature showed that 37 patients were diagnosed following surgical resection, including two patients who underwent incomplete resection and four who were found to have ACA incidentally [4]. One patient was diagnosed by surgical biopsy [1, 8].

ERP of the presented case clearly described that an image of MPD which is not connected to any cyst or without stenosis differs from images common to IPMN and other malignant tumors. Furthermore, small branches of the pancreatic duct were detected, suggesting that the tumor was soft and plastic. Surgical findings were also in agreement, providing further evidence for acinar metaplasia. Relative to MRP, which most previous reports presented, ERP can provide more precise information on the pancreatic duct without interference by cystic lesions. These finding may be useful in distinguishing ACAs from other cystic neoplasms, although it is not definitive.

Histologically, the surface of the cystic walls consists of one or several cell layers with normal acinar cells; cuboidal cells with round, basally oriented nuclei and eosinophilic, periodic acid stain (PAS)-positive granules in its apical cytoplasm. Mitoses are rarely observed [2, 4, 11]. In terms of immunohistochemistry, the staining patterns are identical to those of normal acinar cells of the pancreas, expressing pancreatic enzymes, positive at chymotrypsin, trypsin, lipase, and epithelial markers such as AE1/AE3 and CAM-5.2. The expression of CK7 was reported to be an identifiable difference from non-neoplastic acinar cells [4, 7, 10]. Usually, MIB-1 index is <1 % [4].

The pathogenesis of ACAs remains unclear. Now, it is assumed that acinar metaplasia could be the main pathogenesis of ACAs based on results of the immunohistochemistry [2]. However, Khor et al. have suggested that ACAs are neoplastic because of the augmentation in copy number of specific genes, which array comparative genomic hybridization provides in an ACA with mural nodules [7]. In contrast, the polyclonality of the X chromosome suggested that ACAs are benign non-neoplastic ballooning of the acinar epithelium [10]. Concerning malignant potential or transformation of ACAs to acinar cell carcinoma, there also is insufficient evidence to determine factors associated with it [4]. Although the WHO classification also does not describe a relationship between ACAs and acinar cell carcinoma [15], this relationship has not yet been determined because ACAs are newly recognized entities and ACAs, acinar cell carcinomas, and acinar cystadenocarcinomas are very rare.

At present, although ACAs are finally diagnosed by pathological examination of surgical specimen, we would like to propose two approaches when you encounter cystic lesions. First, the diagnostic clues of ACAs can be proposed from our experience and the literatures as follows: younger females, round-shaped cystic tumor without evidence of the MPD stricture, no communications to the MPD (unlike IPMN), thin capsules (unlike MCN), and non-honeycomb type (unlike SCN). Second, modification of the general approach may be preferable when the ACAs are included in the differential diagnosis of the cystic mass. Occasionally, total pancreatectomy or pancreatoduodenectomy are required for resection when the lesions, suspected of being malignant, are multiple, diffuse, or located in the pancreatic head. In such situations, surgical biopsy, which is not included in the guidelines for the management of IPMN and MCN [14], can be an option of diagnosing strategy.

## Conclusions

Here, we reported a rare surgical case of ACA following a long-term surveillance. It showed interesting radiologic features and clinical courses. Clinicians are recommended to consider an ACA as one of differential diagnoses of cystic tumors of the pancreas to provide an appropriate treatment.

### Consent

Written informed consent was obtained from the patient for publication of this case report and any accompanying images. A copy of the written consent is available for review by the Editor-in-Chief of this journal.

## Abbreviations

ACA: acinar cell cystadenoma
BD-IPMN: branch duct intraductal papillary mucinous neoplasms
CT: computed tomography
ERP: endoscopic retrograde pancreatography
EUS: endoscopic ultrasound sonography
MCN: mucinous cystic neoplasm
MPD: main pancreatic duct
MRI: magnetic resonance imaging
PAS: periodic acid stain
SCN: serous cystic neoplasm
